# Hydration, Fluid Intake, and Related Urine Biomarkers among Male College Students in Cangzhou, China: A Cross-Sectional Study—Applications for Assessing Fluid Intake and Adequate Water Intake

**DOI:** 10.3390/ijerph14050513

**Published:** 2017-05-11

**Authors:** Na Zhang, Songming Du, Zhenchuang Tang, Mengqi Zheng, Ruixia Yan, Yitang Zhu, Guansheng Ma

**Affiliations:** 1 Department of Nutrition and Food Hygiene, School of Public Health, Peking University, 38 Xue Yuan Road, Haidian District, Beijing 100191, China; ziqingxuanping@126.com; 2 Beijing Key Laboratory of Toxicological Research and Risk Assessment for Food Safety, School of Public Health, Peking University, 38 Xue Yuan Road, Haidian District, Beijing 100191, China; 3 Chinese Nutrition Society, 6 Guang An Men Nei Street, Xicheng District, Beijing 100053, China; dusm9709@126.com; 4 Institute of Food and Nutrition Development, Ministry of Agriculture, 12 Zhong Guan Cun Nan Street, Haidian District, Beijing 100181, China; tangzhenchuang@126.com; 5 National Institute for Nutrition and Health, Chinese Center for Disease Control and Prevention, 29 Nan Wei Road, Xicheng District, Beijing 100050, China; zhengmq93@163.com; 6 Department of Health Management and Service, Cangzhou Medical College, Higher Education District, Cangzhou 061000, China; yrx2002@sina.com; 7 Clinical Laboratory, Cangzhou Central Hospital, Xinhua District, Cangzhou 061000, China; zhuyitangcz@126.com

**Keywords:** fluid intake, hydration, urine osmolality

## Abstract

The objectives of this study were to assess the associations between fluid intake and urine biomarkers and to determine daily total fluid intake for assessing hydration status for male college students. A total of 68 male college students aged 18–25 years recruited from Cangzhou, China completed a 7-day cross-sectional study. From day 1 to day 7; all subjects were asked to complete a self-administered 7-day 24-h fluid intake record. The foods eaten by subjects were weighed and 24-h urine was collected for three consecutive days on the last three consecutive days. On the sixth day, urine osmolality, specific gravity (USG), pH, and concentrations of potassium, sodium, and chloride was determined. Subjects were divided into optimal hydration, middle hydration, and hypohydration groups according to their 24-h urine osmolality. Strong relationships were found between daily total fluid intake and 24-h urine biomarkers, especially for 24-h urine volume (*r* = 0.76; *p* < 0.0001) and osmolality (*r* = 0.76; *p* < 0.0001). The percentage of the variances in daily total fluid intake (*R*^2^) explained by PLS (partial least squares) model with seven urinary biomarkers was 68.9%; two urine biomarkers—24-h urine volume and osmolality—were identified as possible key predictors. The daily total fluid intake for assessing optimal hydration was 2582 mL, while the daily total fluid intake for assessing hypohydration was 2502 mL. Differences in fluid intake and urine biomarkers were found among male college students with different hydration status. A strong relationship existed between urine biomarkers and fluid intake. A PLS model identified that key variables for assessing daily total fluid intake were 24-h urine volume and osmolality. It was feasibility to use total fluid intake to judge hydration status.

## 1. Introduction

Water is the main component of the human body and is essential for human survival. Water plays important roles in body metabolism, maintaining electrolyte balance, modulating normal osmotic pressure, and regulating body temperature. Optimal hydration status is important for maintaining health. Both hyperhydration and hypohydration have negative impacts on health. If fluid intake exceeds the capacity of renal excretion (700–1000 mL/h) [1], it can result in acute water intoxication, and even hyponatremia. Water intoxication, along with the symptoms of brain cell swelling, cerebral edema, and increased intracranial pressure, can result in headache, nausea, and memory loss, even progressive mental retardation, trance, coma, convulsions, and death. Hypohydration occurs when the fluid intake is insufficient to replace the free water output. It has been reported that hypohydration may impair cognitive performances, such as short-term memory [2,3], psychomotor skills, vigilant attention, choice reactions, and perceptive discrimination [4]. Hypohydration also reduced the ability to perform physical activities [5,6]. In addition, hypohydration increased the incidence and the prevalence of urinary system diseases [7,8,9,10]. A clear relationship between the hardness of water and cardiovascular diseases was also reported [11,12,13]. It is crucial to maintain optimal hydration in order to ensure the normal functions of human body.

A few methods can be applied to assess hydration status, including serum osmolality, urine volume, urine osmolality, and other urine biomarkers. Serum osmolality has been suggested as a good marker for acute hypohydration but was insensitive to mild hypohydration with ad libitum access to fluid [14]. Urine biomarkers reflecting total fluid intake [15] were useful and biologically significant for assessing mild hypohydration [16,17]. Some studies have reported that 24-h urine biomarkers were strongly correlated with daily total fluid intake [18,19,20]. However, few studies about the association between fluid intake and related biomarkers have been conducted in China.

Only two surveys regarding fluid intake were carried out in 2010 and 2011. One survey for assessing fluid intake among 1483 adults from four cities in China found that approximately 32% of the subjects drank less fluid than the amount recommended by the Chinese Nutrition Society in 2007 (1200 mL/day). Another survey for assessing fluid intake among 5868 primary and middle school students from four cities reported that nearly two-thirds of the subjects drank less than the recommended amount in 2007 (1200 mL/day). A large proportion of Chinese residents may be in hypohydration status [21]. This requires additional study in China due to the following reasons. The behaviors of fluid intake differ among Chinese, Europeans, and Americans. Chinese people prefer tea, while Europeans and Americans tend to drink more coffee [22]. In addition, fluid intake from food was different among China and some other countries [23]. In Europe and the United States, the proportion of fluid intake from food accounts for about 20% of total fluid intake [24], whereas in China, the proportion of fluid intake from food accounts for nearly 44% [25]. In addition, the landscapes vary significantly across its vast area, and the climates vary in different regions due to the highly complex topography in China [26]. Heights are also different among the people of China and other countries due to race differences [27], which contributes to difference in body surface area and has corresponding effects on water requirements [28]. Thus, although adequate water intake has been suggested in some countries, it is still necessary to recommend specific adequate water intake in China with full consideration of hydration and other factors.

The aims of this study are: firstly, to compare the differences of urine biomarkers among subjects in optimal hydration (24-h urine osmolality ≤ 500 mOsm/kg), middle hydration (500 Osm/kg < 24-h urine osmolality ≤ 800 Osm/kg), and hypohydration (24-h urine osmolality > 800 mOsm/kg) [1,29]; secondly, to explore the associations between fluid intake and urine biomarkers; and finally, to determine the daily total fluid intake for assessing optimal hydration and hypohydration status.

## 2. Materials and Methods

### 2.1. Subjects

Subjects were recruited from the freshmen and sophomores of a medical college in Cangzhou, Hebei province, China.

Inclusion criteria: Subjects aged between 18 and 25 years with good health status were included.

Exclusion criteria: Subjects were excluded for tobacco use, or habitual high alcohol (>20 g/day) consumption, or intensive physical exercise habits, or cognitive disorders and other diseases (e.g., diabetes, gastrointestinal tract disease, oral disease, kidney disease, or other chronic or metabolic diseases) [30].

### 2.2. Ethics

The study protocol and instruments were reviewed and approved by the Ethical Review Committee of the Chinese Nutrition Society in 10 December 2015 (project identification code: CNS-2015-001). The study was carried in compliance with the guidelines of the Declaration of Helsinki. All the subjects read and signed the informed consent form prior to the study.

### 2.3. Study Design and Procedure

**Figure 1:**
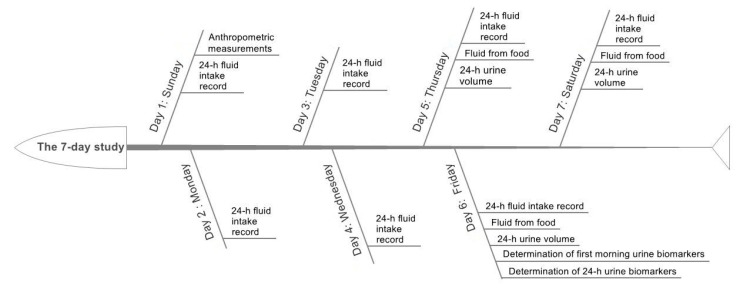
The study procedure.

A 7-day cross-sectional study was designed and performed; see Figure 1.

On the first day, the height, weight, waist circumference, body protein content, and percent body fat mass of subjects were measured before breakfast following a standardized procedure by trained staff. In order to assess the daily total fluid intake from water and other beverages, all subjects were asked to complete a self-administered 7-day 24-h fluid intake record after training to use the fluid intake record correctly. The foods eaten by subjects were weighed on three consecutive days (including two weekdays and one weekend day) during the seven days, with the aim of assessing the daily fluid intake from food. The 3-day 24-h urine of the subjects was collected to measure the 24-h urine volume. On the sixth day, 24-h urine was collected to determine the urine osmolality, urine-specific gravity, pH, and the concentrations of potassium, sodium, and chloride. The outdoor and indoor temperature and humidity in the study site were recorded at 10:00 a.m., 2:00 p.m., and 8:00 p.m. each day during the seven days.

### 2.4. Anthropometric Measurements

Height and weight were measured by trained investigators with a height-weight meter (HDM-300; Huajun, Zhejiang, China). Height was measured twice to the nearest 0.1 cm in bare feet. Fasting body weight was measured twice to the nearest 0.1 kg with the subjects wearing light clothing. Averages for height and weight were calculated.

Waist circumference was measured by trained investigators with a MyoTape waistline measurer (Accu Measure, Greenwood Village, CO, USA). Waist circumference was measured twice to the nearest 0.1 cm with the subjects standing and being free of clothing covering the waist area. Waist circumstance was measured at the midpoint between the bottom of the rib cage and the top of the iliac crest at the end of exhalation. Average for waist circumference was calculated.

Body protein content and percent body fat mass (%) were measured by trained investigators with a body composition analyzer (Inbody 720; Inbody; Seoul, Korea) [31].

Body Mass Index (BMI) = weight (kg)/height (m)^2^

Body surface area (m^2^) = weight (kg)^0.425^ × height (cm)^0.725^ × 0.007184 [32].

### 2.5. Assessment of Fluid Intake

The information on daily total drinking fluid of the subjects was collected using a 7-day 24-h fluid intake record that was recorded by themselves. Subjects were trained to use the fluid intake record correctly. The amount of drinking fluid for each time was measured using a cup to the nearest of 10 mL. Subjects poured fluid into the cups to measure the amount before they drank bottled water, sugar sweetened beverages, milk, etc. The 7-day 24-h fluid intake record has been used in previous studies and has been proven to be reliable [21].

The daily fluid intake from food: all food consumed by the subjects were weighed for three consecutive days (including two weekdays and one weekend day) in order to assess daily fluid intake from food with a duplicate portion method. The fluid intake from food was measured according to GB 5009.3-2010 [33]. The fluid from fruits was assessed by trained investigators using the China Food Composition Table [34].

Daily total fluid intake (mL) = Daily total drinking fluid (mL) + Daily fluid intake from food (mL).

### 2.6. Assessment of Urine Biomarkers

Urine samples starting with the second voiding of the day and ending with the first voiding the following morning were collected in disposable flexible packaging plastic bags by subjects [35]. Urine samples were stored at 4 °C in a refrigerator prior to assessments.

Urine volume was measured to the nearest 0.1 kg using a desktop electronic scale (YP20001, SPC, Shanghai, China). Urine osmolality was tested using an osmotic pressure molar concentration meter (SMC 30C, Tianhe, Tianjin, China) with the freezing-point method. Urine-specific gravity (USG) was determined using an automatic urinary sediment analyzer (FUS-200, Dirui, Changchun, China) with the uric dry-chemistry method. Urine pH was measured using a urine analyzer (Uritest-180, Uritest, Guilin, China) with the reflective photoelectric colorimetry method. The urine concentrations of potassium, sodium, and chloride were determined using an automatic biochemical analyzer (Cobas C501, Roche, Basel, Switzerland) with the ion-selective electrode potentiometer method.

### 2.7. Temperature and Humidity of the Environment

The temperature and humidity both indoors and outdoors at the study site were measured using a temperature hygrometer (WSB-1-H2, Exasace, Zhengzhou, China) each day during the seven study days.

### 2.8. Statistical Analyses

SAS 9.2 (SAS Institute, Inc., Cary, NC, USA) was used for the statistical analyses. Quantitative data are presented as mean ± standard deviation (SD), numeration data were presented as *n* (percentage). Fluid intake was an average for seven days; urine volume was an average for three days; other urine biomarkers were the actual value for one day. Subjects were divided into optimal hydration, middle hydration, and hypohydration groups according to their 24-h urine osmolality. The differences of quantitative data among the different hydration statuses were analyzed with ANOVA, while the differences in numeration data were analyzed by χ^2^ test. Pearson’s correlation coefficients were performed to determine the strength of the relationship between fluid intake and related 24-h urine biomarkers. A multivariable partial least squares (PLS) model was used to identify the key predictors in modeling the daily total fluid intake with 24-h urine biomarkers. A total of seven urinary biomarkers (urine volume, osmolality, specific gravity, pH, and concentrations of K, Na, and Cl) were considered to be predictors of total fluid intake. Binary variables were established based on 24-h urine osmolality, which was used to assess optimal hydration (0: optimal hydration; 1: middle hydration + hypohydration) and assess hypohydration (0: optimal hydration + middle hydration; 1: hypohydration), respectively. Logistic regressions of daily total fluid intake against binary variables were performed, and receiver operating characteristic curve (ROC) analyses were analyzed to determine the cutoff value of daily total fluid intake for optimal hydration and hypohydration status without adjustments to favor either sensitivity or specificity. Significance level was set at 0.05 (*p* < 0.05).

## 3. Results

### 3.1. Characteristics of the Subjects and Environment

A total of 68 subjects were recruited and completed the study with a 100% completion rate. Twenty-two subjects were classified into the optimal hydration group, whereas 27 subjects and 17 subjects were classified into the middle hydration group and the hypohydration group according to their 24-h urine osmolality, respectively. No statistically significant differences were found in age, height, weight, Body Mass Index (BMI), waist circumference, body surface area, body protein content, and percent body fat mass among the three groups with different hydration status. The characteristics of the subjects are shown in Table 1.

### 3.2. Temperature and Humidity

The average indoor and outdoor temperature for the seven days was 14.4 °C ± 1.6 °C and 8.3 °C ± 3.0 °C, respectively, while the average indoor and outdoor humidity was 32.6% ± 1.2% and 35.0% ± 3.4%, respectively (Table 2). The average time that the subjects spent outdoors and indoors was 4 h and 20 h for workdays, and 8 h and 16 h for days occurring on the weekend, respectively.

### 3.3. Assessment of Fluid Intake

Statistically significant differences were found in daily total fluid intake, daily fluid intake from food, and daily total drinking fluid among the three groups with different hydration status (*F* = 28.956, *p* = 0.000; *F* = 15.628, *p* = 0.000; *F* = 27.410, *p* = 0.000). The percentage of individuals meeting adequate total fluid intake and adequate total drinking fluid intake (according to the intake recommendations in China) were also statistically significantly different among the three groups (*F* = 22.667, *p* = 0.000; *F* = 20.065, *p* = 0.000). Subjects in the optimal hydration group had significantly more daily total fluid intake, daily fluid intake from food, and daily total drinking fluid than their counterparts in both the middle hydration group and the hypohydration group. Significantly more subjects in the optimal hydration group met adequate total fluid intake and adequate total drinking fluid intake (according to the intake recommendations in China) than those in the other two hydration groups. Subjects in the middle hydration group had significantly more daily total fluid intake and daily total drinking fluid than subjects in hypohydration group (Table 3).

### 3.4. Assessment of Urine Biomarkers

Statistically significant differences in 24-h urine volume, 24-h urine osmolality, 24-h urine USG, 24-h potassium, 24-h sodium and 24-h chloride were found among the three groups (*F* = 15.621, *p* = 0.000; *F* = 183.120, *p* = 0.000; *F* = 29.127, *p* = 0.000; *F* = 37.169, *p* = 0.000; *F* = 62.393, *p* = 0.000; *F* = 66.222, *p* = 0.000, respectively). Subjects in the optimal hydration group had significantly more 24-h urine volume and lower 24-h urine osmolality, 24-h USG, and concentrations of 24-h potassium, 24-h sodium and 24-h chloride than those in the middle hydration and hypohydration group. Subjects in the middle hydration group had significantly more 24-h urine volume and lower 24-h urine osmolality, 24-h USG, and concentrations of 24-h potassium, 24-h sodium and 24-h chloride than those in the hypohydration group (Table 4).

### 3.5. Association between Fluid Intake and 24-h Urine Biomarkers

Strong relationships were found between 24-h urine biomarkers and the daily total fluid intake, especially for the 24-h urine volume (*r* = 0.76, *p* < 0.0001) and osmolality (*r* = 0.76, *p* < 0.0001) (Table 5).

### 3.6. Partial Least Squares Model of the Relationship between Daily Total Fluid Intake and 24-h Urine Biomarkers

A PLS model of the relationship between daily total fluid intake and 24-h urinary biomarkers was developed using seven urinary measurements as variables. The percentage of variance in daily total fluid intake (*R*^2^) explained by the PLS model was 68.9%, with a root mean square error of 364 mL. In the PLS model, two urine biomarkers—24-h urine volume and osmolality—were identified as possible key predictors of daily total fluid intake. The 24-h urine volume and osmolality contributed most heavily to the PLS model, with a variable importance in projection (VIP) of 1.41 and 1.17, respectively (Table 6).

**Figure 2:**
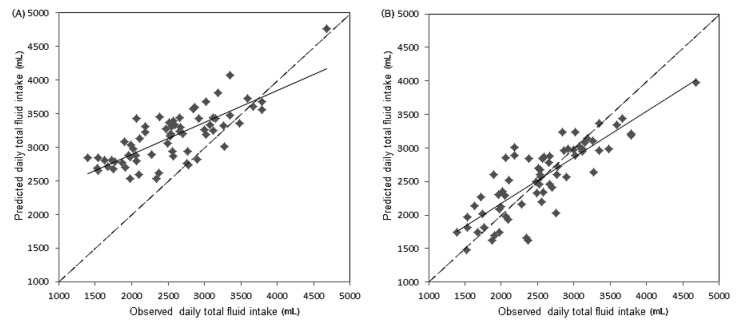
PLS model of the relationship between daily total fluid intake and urine biomarkers. (**A**) PLS model of the relationship between daily total fluid intake and seven variables. The solid line represents the line of agreement, while the dashed line represents the line of best agreement. (**B**) PLS model of the relationship between daily total fluid intake and two variables—urine volume and osmolality. The solid line represents the line of agreement, while the dashed line represents the line of best agreement.

In the PLS model with the 24-h urine volume and osmolality as variables, the percentage of variance in daily total fluid intake (*R*^2^) explained by the PLS model was 67.7%, with a root mean square error of 370 mL (Figure 2).

### 3.7. Determination of the Daily Total Fluid Intake for Assessing Optimal Hydration and Assessing Hypohydration

**Figure 3:**
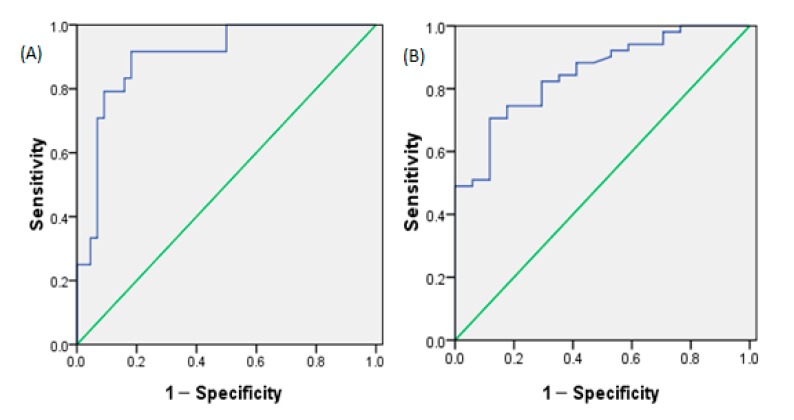
Receiver operating characteristic curve (ROC) analysis curve of the daily total fluid intake for assessing optimal hydration and assessing hypohydration. (**A**) ROC for assessing optimal hydration. (**B**) ROC for assessing hypohydration.

The daily total fluid intake for assessing optimal hydration was 2582 mL (area under the curve = 0.895), with sensitivity 91.7% and specificity 81.8%. The daily total fluid intake for assessing hypohydration was 2502 mL (area under the curve = 0.848), with sensitivity 70.6% and specificity 88.2% (Figure 3).

## 4. Discussion

The balance between water output and water input defines hydration. Hydration is the condition of healthy individuals who maintain a water balance. “Desirable urine osmolality” was defined as 500 mOsm/kg by European Food Safety Authority (EFSA), a definition that is consistent with the cut-off value calculated by Perrier et al. [30]. People are considered as being in optimal hydration status when urine osmolality is ≤500 mOsm/kg. Optimal hydration means that daily fluid intake is sufficient to compensate fluid loss and maintain normal urine volume and is helpful for reducing the risk of related chronic diseases, such as urolithiasis, cardiovascular diseases, and so on [30]. People are considered as being in hypohydration status when urine osmolality is >800 mOsm/kg [36]. Hypohydration occurs when fluid intake is insufficient to replace the water lost due to physiological processes. In this study, subjects were divided into three groups: optimal hydration group, middle hydration group, and hypohydration group according to their 24-h urine osmolality. As expected, subjects in the optimal hydration group had more fluid intake and 24-h urine volume, and lower 24-h urine osmolality, specific gravity, potassium, sodium, and chloride concentrations in this study.

To further study the relationship among daily total fluid intake and 24-h urine biomarkers, it was found that almost all urine biomarkers in the study were associated with daily total fluid intake among male college students, especially for 24-h urine volume and osmolality. The results were consistent with those of Perrier et al., who suggested that strong associations (|*r*| ≥ 0.6) existed between total fluid intake and the 24-h urine volume, osmolality, specific gravity, and solute concentrations [16]. McKenzie et al. also reported a significant relationship between total fluid intake and urine volume in pregnant and lactating women (*r* = 0.71) [37]. Similarly, Perrier et al. concluded that subjects who had ingested low amounts of fluid had smaller amounts of more concentrated urine [17]. In the study of McKenzie et al., urinary hydration biomarkers also had a strong relationship with total fluid intake in pregnant and breast-feeding women [19]. In this study, a PLS model of the relationship between daily total fluid intake and 24-h urinary biomarkers was developed. However, the root mean square error was 364 mL, which represents a certain degree of inaccuracy. In a similar model developed by Perrier et al., the root mean square error was 663 mL [16]. In another study of Perrier et al., it was suggested that 24-h urine osmolality was a physiological index of adequate fluid intake [38]. Logistic regression of the daily total fluid intake against a binary hydration outcome was performed. The results suggested that the daily total fluid intake for assessing hypohydration (urine osmolality > 800) was 2502 mL among male college students, which was close to the amount of fluid intake recommended by ESFA. Armstrong et al. also concluded that urine osmolality could be used to determine total fluid intake and urine osmolality of 800 mOsm/kg corresponded to a total fluid intake of ≥2.4 L/day, which was similar to the conclusion in our study [38]. Furthermore, the daily total fluid intake for assessing optimal hydration was also determined and was 2582 mL in our study, which may be more helpful for keeping health and reducing the risk of hydration-related diseases.

There were some strengths and weaknesses in our study. In terms of strengths, this was the first study to explore the relationship between fluid intake and urine biomarkers and the optimal fluid intake for assessing optimal hydration based on urine osmolality in China. The cross-sectional survey was carried out among subjects in free-living conditions. Temperature and humidity both have effects on fluid intake. Under high temperature conditions, water evaporation from the surface of the body’s skin increases and the body is prone to hypohydration. Humidity mainly has effects on the processes of perspiration and the processes of metabolism associated with water and electrolytes. Thus, in this study, temperature and humidity were also recorded. In terms of weaknesses, however, these results require replication. Subjects with different genders and ages were not investigated in our study. The urine biomarkers used were not comprehensive enough, and plasma hydration biomarkers were not explored.

This study is of great significance for the applications in two following respects. First, it provides the possibility of assessing fluid intake based on urine biomarkers instead of the traditional and complex fluid intake surveys. In previous studies, complex diet compositions, large food samples, and expensive laboratory testing costs added difficulties to the survey of fluid intake from foods. There are also some weaknesses in the seven-days 24-h fluid intake recording methods: poor compliance and poor accuracy. Establishing a model based on urine biomarkers may be another meaningful way to assess fluid intake. In another respect, the study provides more evidence for revising adequate fluid intake guidelines. Although some countries and organizations have proposed fluid intake recommendations, the optimal amount of fluid intake to maintain optimal hydration has not been fully considered in China. Future studies should explore the relationship between fluid intake and urine biomarkers with larger samples of different ages and genders to obtain more accurate data with comparability. Adequate fluid intake guidelines should be continuously improved, with full consideration of hydration.

## 5. Conclusions

In conclusion, fluid intake and urine biomarkers were different among male college students with different hydration status in this study. Strong relationships were found between 24-h urine biomarkers and daily total fluid intake, especially for 24-h urine volume and osmolality among male college students. There is a certain feasibility associated with using fluid intake for assessing hydration status. The optimal daily total fluid intake for assessing optimal hydration and assessing hypohydration was 2582 mL and 2502 mL among male college students, respectively.

## Figures and Tables

**Table 1:** Characteristics of the subjects with three hydration statuses.

	All Subjects (*n* = 68)	Optimal Hydration (*n* = 24)	Middle Hydration (*n* = 27)	Hypohydration (*n* = 17)	*F*	*p*
Age (years)	19.9 ± 1.1	20.3 ± 1.2	19.7 ± 1.1	19.5 ± 0.8	2.931	0.060
Height (cm)	174.0 ± 5.2	173.1 ± 4.6	174.6 ± 5.3	174.4 ± 6.0	0.594	0.555
Weight (kg)	67.9 ± 10.8	67.0 ± 11.0	67.8 ± 12.2	69.5 ± 8.3	0.249	0.780
BMI (kg/m^2^)	22.4 ± 3.6	22.4 ± 3.5	22.2 ± 4.0	22.9 ± 3.2	0.173	0.842
Waist circumference (cm)	79.2 ± 9.0	79.6 ± 7.9	78.9 ± 10.6	79.2 ± 8.1	0.046	0.955
Body surface area (m^2^)	1.813 ± 0.133	1.796 ± 0.135	1.814 ± 0.148	1.835 ± 0.106	0.430	0.652
Body protein content (kg)	10.7 ± 1.2	10.5 ± 1.2	10.6 ± 1.2	11.0 ± 1.0	1.086	0.343
Percent body fat mass (%)	20.1 ± 6.8	20.7 ± 6.9	19.7 ± 7.4	19.8 ± 5.9	0.144	0.866

Note: Values are shown as the mean ± standard deviation (SD). BMI: Body Mass Index.

**Table 2:** Indoor and outdoor temperature and humidity for the seven study days.

	Indoors		Outdoors	
	Temperature (°C)	Humidity (%)	Temperature (°C)	Humidity (%)
Sunday	15.3	33	11.6	32
Monday	16.9	34	12.5	32
Tuesday	14.6	31	5.4	35
Wednesday	15.2	32	5.6	34
Thursday	13.9	33	6.1	35
Friday	13.1	31	6.9	35
Saturday	12.0	34	10.0	42

**Table 3:** Fluid intake of subjects with three hydration statuses.

	All Subjects (*n* = 68)	Optimal Hydration (*n* = 24)	Middle Hydration (*n* = 27)	Hypohydration (*n* = 17)	*F*	*p*
Daily total fluid intake (mL)	2553 ± 653	3114 ± 543 ^#^	2405 ± 467 ^‡^	1995 ± 407 ^†^	28.956	0.000 *
Percentage meeting adequate total fluid intake (based on intake recommendations in China) (%)	17 (25.0)	14 (58.3) ^#^	3 (6.8)	0 (0.0) ^†^	22.667	0.000 *
Percent meeting the EFSA fluid intake recommendations (%)	38 (55.9)	22 (91.7)	12 (17.6)	7 (10.3) ^†^	3.608	0.165
Daily fluid intake from food (mL)	1211 ± 232	1381 ± 202 ^#^	1155 ± 202	1059 ± 165 ^†^	15.628	0.000 *
Daily total drinking fluid (mL)	1342 ± 468	1733 ± 399 ^#^	1250 ± 342 ^‡^	936 ± 281 ^†^	27.410	0.000 *
Percentage meeting adequate total drinking fluid intake (based on intake recommendations in China) (%)	16 (23.5)	13 (54.2) ^#^	3 (6.8)	0 (0.0) ^†^	20.065	0.000 *

Note: Values are shown as the mean ± SD, with the exception that percentages were shown as *n* (percentage). * There was statistically significant difference among three hydration groups, *p* < 0.05. ^#^ There was statistically significant difference between optimal hydration and middle hydration groups, *p* < 0.017. ^†^ There was statistically significant difference between optimal hydration and hypohydration groups, *p* < 0.017. ^‡^ There was statistically significant difference between hypohydration and middle hydration groups, *p* < 0.017. ESFA: European Food Safety Authority.

**Table 4:** Urine biomarkers of subjects with three hydration statuses.

	All Subjects (*n* = 68)	Optimal Hydration (*n* = 24)	Middle Hydration (*n* = 27)	Hypohydration (*n* = 17)	*F*	*p*
24-h urine volume (mL)	1358 ± 460	1653 ± 420 ^#^	1338 ± 393 ^‡^	975 ± 307 ^†^	15.621	0.000 *
24-h urine osmolality (mOsm/kg)	653 ± 201	452 ± 43 ^#^	657 ± 86 ^‡^	932 ± 104 ^†^	183.120	0.000 *
24-h urine USG	1.011 ± 0.003	1.014 ± 0.003 ^#^	1.019 ± 0.003 ^‡^	1.010 ± 0.003 ^†^	29.127	0.000 *
24-h urine pH	6.5 ± 0.3	6.7 ± 0.4	6.7 ± 0.3	6.9 ± 0.3	2.557	0.085
24-h urine potassium (mmol/L)	27.7 ± 11.8	19.2 ± 5.2 ^#^	26.5 ± 5.2 ^‡^	41.5 ± 13.8 ^†^	37.169	0.000 *
24-h urine sodium (mmol/L)	164 ± 53	127 ± 33 ^#^	152 ± 20 ^‡^	235 ± 43 ^†^	62.393	0.000 *
24-h urine chloride (mmol/L)	142 ± 53	105 ± 27.4 ^#^	129 ± 18.1 ^‡^	215 ± 48.0 ^†^	66.222	0.000 *

Note: Values are shown as the mean ± SD. * There was statistically significant difference among three hydration groups, *p* < 0.05. ^#^ There was statistically significant difference between optimal hydration and middle hydration, *p* < 0.017. ^†^ There was statistically significant difference between optimal hydration and hypohydration, *p* < 0.017. ^‡^ There was statistically significant difference between hypohydration and middle hydration, *p* < 0.017. USG: urine specific gravity.

**Table 5:** Correlations between fluid intake and 24-h urinary biomarkers.

	Daily Total Fluid Intake		Fluid Intake from Food		Daily Total Drinking Water	
	*r*	*p*	*r*	*p*	*r*	*p*
All subjects (*n* = 68)						
24-h urine volume	0.76	0.000 *	0.7	0.000 *	0.72	0.000 *
24-h urine osmolality	−0.76	0.000 *	−0.63	0.000 *	−0.75	0.000 *
24-h USG	−0.56	0.000 *	−0.47	0.000 *	−0.54	0.000 *
24-h pH	−0.25	0.072	−0.19	0.359	−0.25	0.038 *
Potassium (mmol/L)	−0.5	0.000 *	−0.45	0.000 *	−0.47	0.000 *
Sodium (mmol/L)	−0.61	0.000 *	−0.45	0.000 *	−0.62	0.000 *
Chloride (mmol/L)	−0.57	0.000 *	−0.42	0.001 *	−0.59	0.000 *

* Indicates *p* < 0.05.

**Table 6:** Variable importance in projection (VIP) coefficients for 24-h urine hydration biomarkers in the partial least squares (PLS) model.

VIP > 0.8		VIP < 0.8	
24-h urine volume	1.41	24-h urine potassium concentration	0.84
24-h urine osmolality	1.17	24-h urine pH	0.45
24-h urine-specific gravity	0.91		
24-h urine sodium concentration	0.98		
24-h urine chloride concentration	0.98		
